# The share of ultra-processed foods and the overall nutritional quality of diets in the US: evidence from a nationally representative cross-sectional study

**DOI:** 10.1186/s12963-017-0119-3

**Published:** 2017-02-14

**Authors:** Euridice Martínez Steele, Barry M. Popkin, Boyd Swinburn, Carlos A. Monteiro

**Affiliations:** 1 Department of Nutrition, School of Public Health, University of São Paulo, Av. Dr. Arnaldo, 715, 01246-907 São Paulo, Brazil; 2 Center for Epidemiological Studies in Health and Nutrition, University of São Paulo, São Paulo, Brazil; 3 Department of Nutrition, University of North Carolina at Chapel Hill, Chapel Hill, NC USA; 4 School of Population Health, University of Auckland, Auckland, New Zealand

**Keywords:** NHANES, Ultra-processed, Dietary nutrient profile, PCA, Dietary patterns, Diet quality, Macronutrients, Micronutrients

## Abstract

**Background:**

Recent population dietary studies indicate that diets rich in ultra-processed foods, increasingly frequent worldwide, are grossly nutritionally unbalanced, suggesting that the dietary contribution of these foods largely determines the overall nutritional quality of contemporaneous diets. Yet, these studies have focused on individual nutrients (one at a time) rather than the overall nutritional quality of the diets. Here we investigate the relationship between the energy contribution of ultra-processed foods in the US diet and its content of critical nutrients, individually and overall.

**Methods:**

We evaluated dietary intakes of 9,317 participants from 2009 to 2010 NHANES aged 1+ years. Food items were classified into unprocessed or minimally processed foods, processed culinary ingredients, processed foods, and ultra-processed foods. First, we examined the average dietary content of macronutrients, micronutrients, and fiber across quintiles of the energy contribution of ultra-processed foods. Then, we used Principal Component Analysis (PCA) to identify a nutrient-balanced dietary pattern to enable the assessment of the overall nutritional quality of the diet. Linear regression was used to explore the association between the dietary share of ultra-processed foods and the balanced-pattern PCA factor score. The scores were thereafter categorized into tertiles, and their distribution was examined across ultra-processed food quintiles. All models incorporated survey sample weights and were adjusted for age, sex, race/ethnicity, family income, and educational attainment.

**Results:**

The average content of protein, fiber, vitamins A, C, D, and E, zinc, potassium, phosphorus, magnesium, and calcium in the US diet decreased significantly across quintiles of the energy contribution of ultra-processed foods, while carbohydrate, added sugar, and saturated fat contents increased. An inverse dose–response association was found between ultra-processed food quintiles and overall dietary quality measured through a *nutrient-balanced-pattern* PCA-derived factor score characterized by being richer in fiber, potassium, magnesium and vitamin C, and having less saturated fat and added sugars.

**Conclusions:**

This study suggests that decreasing the dietary share of ultra-processed foods is a rational and effective way to improve the nutritional quality of US diets.

**Electronic supplementary material:**

The online version of this article (doi:10.1186/s12963-017-0119-3) contains supplementary material, which is available to authorized users.

## Background

Ultra-processed foods are formulations manufactured using several ingredients and a series of processes (hence “ultra-processed”). Most of their ingredients are lower-cost industrial sources of dietary energy and nutrients, and additives used for the purpose of imitating sensorial qualities of minimally processed foods or of culinary preparations of these foods, or to disguise undesirable sensory qualities of the final product. They are made to be hyper-palatable and attractive by the use of many additives, with long shelf life, and are able to be consumed anywhere, anytime. Ultra-processed foods include but are not limited to soft drinks, sweet or savory snacks, reconstituted meat products, and pre-prepared frozen dishes [1–6].

In studies carried out in nationally representative samples of the Brazilian population it has been shown that the group of ultra-processed foods have higher content of free sugars, total fats, saturated fats, and trans fats, and lower content of protein, fiber, and most micronutrients than the rest of the diet, and that high consumption of ultra-processed foods renders grossly nutritionally unbalanced diets [7–9]. In Canada, similar results have been documented regarding free sugars, total fats, protein, and fiber [10]. In the US, using 2009–2010 National Health and Nutrition Examination Survey (NHANES) day 1 data, a positive association was found between the dietary contribution of ultra-processed foods and the dietary content of added sugars [11]. Another US study found that highly processed barcoded consumer packaged foods and beverages, mostly ultra-processed products, are higher in saturated fat, sugar, and sodium contents compared to less-processed foods [12].

Based on the detrimental effects of ultra-processed foods on the dietary content of critical nutrients and taking into account their increasing predominance in global food supplies [3, 6, 13–16], the dietary share of ultra-processed foods, expressed as a percentage of total energy intake, has been proposed [1, 4, 17] and further recognized by the United Nations Food and Agriculture Organization [5], the Pan-American Health Organization [6], and INFORMAS (International Network for Food and Obesity/non-communicable diseases Research, Monitoring and Action Support) [18] as a potentially meaningful determinant of the overall nutritional quality of contemporaneous diets.

In order to further evaluate the influence of the dietary share of ultra-processed foods on the nutritional dietary quality we need to study its relationship with the overall nutrient profile of diets. As several authors have pointed out [19–22], studying nutrients one at a time has a number of drawbacks, which may be overcome by focusing on dietary patterns [19, 23–30]. Yet, to date, population studies assessing the impact of ultra-processed food consumption on the nutritional quality of diets have focused on the dietary content of individual nutrients.

Dietary patterns can be derived using two approaches: a priori or a posteriori [31]. A priori techniques use scoring systems or overall measures of dietary quality based on nutritional variables, generally foods and/or nutrients, in order to assess the degree to which a participant complies with a predefined theoretical dietary pattern, created based on current nutrition knowledge. Empirically derived dietary patterns, on the other hand, are patterns derived a posteriori based on observed dietary intake of the various foods and/or nutrients. While a posteriori derived patterns may not necessarily represent optimal dietary patterns, as they are outcome-independent, a priori techniques are limited by the current knowledge which may generate uncertainty regarding which nutrients and cutoff points to use when generating scores [19].

The objective of this study was to examine the relationship between dietary contribution of ultra-processed foods and the nutritional quality of the US diet through the evaluation of dietary contents of critical nutrients individually and also overall, using dietary pattern analysis.

## Methods

### Data source, population and sampling

We utilized nationally representative data from the 2009–2010 National Health and Nutrition Examination Survey (NHANES), a continuous, nationally representative, cross-sectional survey of non-institutionalized, civilian US residents [32].

The survey included an interview conducted in the home and a subsequent health examination performed at a mobile examination center (MEC). All NHANES examinees were eligible for two 24-h dietary recall interviews. The first dietary recall interview was collected in-person in the MEC while the second was collected by telephone three to ten days later. Dietary interviews were conducted by trained interviewers using the validated [33–35] US Department of Agriculture Automated Multiple-Pass Method.

Among the 13,272 people screened in NHANES in 2009–2010, 10,537 (79.4%) participated in the household interview and 10,253 (77.3%) also participated in the MEC health examination. Of these, 9,754 individuals provided one day of complete dietary intakes, and 8406 provided two days’ worth.

We evaluated 9,317 survey participants aged 1 year and above who had at least one day of 24-h dietary recall data and had not been breast-fed on either of the two days. Data for two recall days were used when available, and one day otherwise. These 9,317 individuals had similar sociodemographic characteristics (gender, age, race/ethnicity, family income, and educational attainment) to the full sample of 10,109 interviewed participants aged 1 year and above (Additional file 1: Table S1).

### Food classification according to processing

We classified all recorded food items (*N =* 280,132 Food Codes) according to Nova, a food classification based on the extent and purpose of industrial food processing [4, 17]. Nova includes four groups: “unprocessed or minimally processed foods” (such as fresh, dry, or frozen fruits or vegetables; packaged grains and pulses; grits, flakes, or flours made from corn, wheat, or cassava; pasta, fresh or dry, made from flour and water; eggs; fresh or frozen meat and fish and fresh or pasteurized milk); “processed culinary ingredients” (including salt, vinegar, oils, fats, sugar, and other substances extracted from foods and used in kitchens to season and cook unprocessed or minimally processed foods and to make culinary preparations), “processed foods” (including pickled vegetables, fruit preserves, salted meat products, canned fish in water or oil, cheeses, artisan-style breads (no additives), and other ready-to-consume products manufactured with the addition of salt, vinegar, sugar, oil, or other substances of culinary use to unprocessed or minimally processed foods), and “ultra-processed foods.”

The Nova group of ultra-processed foods, of particular interest in this study, includes soft drinks, sweet or savory packaged snacks, confectionery and industrialized desserts, mass-produced packaged breads and buns, poultry and fish nuggets and other reconstituted meat products, instant noodles and soups, and many other ready-to-consume formulations of several ingredients. Besides salt, sugar, oils, and fats, ultra-processed foods ingredients include food substances not commonly used in culinary preparations, and this is what distinguishes them from processed foods. These ingredients include modified starches, hydrogenated oils, protein isolates, and additives whose purpose is to imitate sensorial qualities of unprocessed or minimally processed foods and their culinary preparations, or to disguise undesirable qualities of the final product, such as colorants, flavorings, non-sugar sweeteners, emulsifiers, humectants, sequestrants, and firming, bulking, de-foaming, anti-caking, and glazing agents. Unprocessed or minimally processed foods represent a small proportion of, or are even absent from, the list of ingredients of ultra-processed products. A detailed definition of each Nova food group and examples of food items classified in each group are shown elsewhere [11]. The rationale underlying the classification is also explained elsewhere [1–3, 36, 37].

For all food items (Food Codes) judged to be a handmade recipe (prepared from fresh or minimally processed foods and processed culinary ingredients), the classification was applied to the underlying ingredients (Standard Reference Codes -SR Codes-) obtained from the USDA Food and Nutrient Database for Dietary Studies (FNDDS) 5.0 [38]. More details in this regard have been previously published [11].

### Assessing energy and nutrient contents

For this study, we used Food Code nutrient values as provided by NHANES.

For handmade recipes, we calculated the underlying ingredient (SR Code) nutrient values using variables from both *FNDDS 5.0* [38] and *USDA National Nutrient Database for Standard Reference, Release 24 (SR24)* [39].

The following nutrients were considered in this study: protein, carbohydrates, added sugars, fats, saturated fats, sodium, vitamins A (as retinol activity equivalents), C, D, and E (as alpha-tocopherol), iron, zinc, potassium, phosphorus, magnesium, calcium, and fiber. These included most underconsumed (vitamins A, C, D, and E, calcium, magnesium, potassium, and fiber) and all overconsumed (sodium, added sugar, and saturated fat) nutrients in the US population [40].

Data on added sugars per Food Code and per SR Code were obtained by merging the Food Patterns Equivalents Database (FPED) 2009–2010 and Food Patterns Equivalents Ingredients Database (FPID) 2009–2010 [41].

We used the following conversion factors: 4 kcal/g for carbohydrates and protein, 9 kcal/g for fat and 7 kcal/g for alcohol. Total energy intake was calculated as the sum of calories from carbohydrates, proteins, fat, and alcohol.

### Data analysis

We utilized all available dietary intake data for each participant, using means of both recall days when available (86% of participants) and one day otherwise.

Food items were sorted into mutually exclusive food subgroups within each of the four Nova groups, as shown in Table 1. First, we evaluated the contributions of each food group and subgroup to total energy intake and across quintiles of the dietary energy contribution of ultra-processed foods (henceforth “dietary share of ultra-processed foods”). The group of unprocessed or minimally processed foods was also combined with the group of processed culinary ingredients, as foods belonging to these two groups are usually combined together in culinary preparations and therefore consumed together.

**Table 1:** Distribution (%) of the total daily per capita energy intake (kcal) according to NOVA food groups by quintiles of the dietary share of ultra-processed foods, US population aged 1+ years (NHANES 2009–2010) (*N =* 9,317)

		Quintile of dietary share of ultra-processed foods (% of total energy intake)^a^				
	All quintiles (*n =* 9,317) (2,069.9 kcal)	Q1 (*n =* 1,941) (1,970.9 kcal)	Q2 (*n =* 1,903) (2,017.6 kcal)	Q3 (*n =* 1,791) (2,061.8 kcal)	Q4 (*n =* 1,785) (2,151.5 kcal)	Q5 (*n =* 1,897) (2,147.7 kcal)
Unprocessed or minimally processed foods	*30.2*	*48.3*	*36.7*	*29.4*	*23.3*	13.2*
Meat (includes poultry)	8.0	11.6	9.6	8	6.7	4*
Fruit and freshly squeezed fruit juices	5.5	8.8	6.8	5.4	4.3	2.5*
Milk and plain yogurt	5.1	6.4	6.1	5.3	4.8	2.9*
Grains	2.9	6.3	3.4	2.3	1.6	0.7*
Roots and tubers	1.7	2.6	2.3	1.7	1.2	0.7*
Eggs	1.5	2.1	1.8	1.4	1.2	0.7*
Pasta	1.4	2.4	1.6	1.4	1.1	0.5*
Legumes	0.9	1.8	1.1	0.8	0.5	0.2*
Fish and seafood	0.8	1.5	1	0.7	0.4	0.2*
Vegetables	0.9	1.5	1	0.8	0.6	0.4*
Other unprocessed or minimally processed foods^b^	1.7	3.2	2	1.5	1	0.5*
Processed culinary ingredients	*2.9*	*4.9*	*3.4*	*2.9*	*2.2*	1.2*
Sugar^c^	1.1	1.6	1.3	1.1	0.9	0.6*
Plant oils	1.2	2.5	1.4	1.2	0.7	0.3*
Animal fats^d^	0.5	0.7	0.6	0.6	0.5	0.2*
Other processed culinary ingredients^e^	0.05	0.1	0.04	0.05	0.03	0.01
Unprocessed or minimally processed foods + Processed culinary ingredients	*33.1*	*53.2*	*40.1*	*32.4*	*25.4*	14.5*
Processed foods	*9.3*	*14.1*	*11.2*	*9.2*	*7.2*	4.8*
Cheese	3.6	4.1	4.1	3.9	3.4	2.5*
Ham and other salted, smoked, or canned meat or fish	1.2	1.5	1.4	1.4	1.1	0.8
Vegetables and other plant foods preserved in brine	0.7	0.9	0.8	0.7	0.6	0.5*
Other processed foods^f^	3.7	7.6	4.8	3.2	2.1	1*
Ultra-processed foods	*57.5*	*32.6*	*48.6*	*58.4*	*67.3*	80.7*
Breads	9.5	7.2	9.9	10.3	10.6	9.4*
Soft and fruit drinks^g^	6.9	3	4.7	6.7	8.2	11.8*
Cakes, cookies, and pies	5.5	2.6	4.6	5.5	6.8	7.9*
Salty snacks	4.4	2.4	3.7	4.3	5.4	6.2*
Frozen and shelf-stable plate meals	3.9	1.3	2.2	3.7	5.2	7.3*
Pizza (ready-to-eat/heat)	3.3	0.5	1.4	2.6	4.1	7.8*
Breakfast cereals	3.1	2.2	3.2	3.6	3.5	3.1
Sauces, dressings, and gravies	2.5	2.4	2.7	2.7	2.8	2.1
Reconstituted meat or fish products	2.3	0.9	2.1	2.4	2.9	2.9*
Ice cream and ice pops	2.3	1.1	1.9	2.4	2.9	3*
Sweet snacks	2.3	1.1	2.1	2.4	2.7	3.4*
Milk-based drinks	1.9	1.1	1.7	1.9	2.1	2.6*
Desserts^h^	1.8	1.3	1.9	2.1	2.1	1.8*
French fries and other potato products	1.7	0.4	1.1	1.7	1.9	3.5*
Sandwiches and hamburgers on bun (ready-to-eat/heat)	1.4	0.2	0.5	1.2	1.5	3.5*
Instant and canned soups	0.9	0.7	0.8	0.9	0.9	1
Other ultra-processed foods^i^	3.8	3.9	4	3.9	3.7	3.2
Total	100.0	100.0	100.0	100.0	100.0	100.0

^a^Mean (range) dietary share of ultra-processed foods per quintile: 1st = 32.6 (0 to 42.6); 2nd = 48.6 (42.6 to 54.0); 3rd = 58.4 (54.0 to 62.8); 4th = 67.3 (62.8 to 72.3); 5th = 80.7 (72.3 to 100)

^b^Including nuts and seeds (unsalted); yeast; dried fruits (without added sugars) and vegetables; non pre-sweetened, non-whitened, non-flavored coffee and tea; coconut water and meat; homemade soup and sauces; flours; tapioca

^c^Including honey, molasses, maple syrup (100%)

^d^Including butter, lard, and cream

^e^Including starches; coconut and milk cream; unsweetened baking chocolate, cocoa powder, and gelatin powder; vinegar; baking powder and baking soda

^f^Including salted or sugared nuts and seeds; peanut, sesame, cashew, and almond butter or spread; beer and wine

^g^Including energy drinks, sports drinks, nonalcoholic wine

^h^Including ready-to-eat and dry-mix desserts such as pudding

^i^Including soy products such as meatless patties and fish sticks; baby food and baby formula; dips, spreads, mustard, and catsup; margarine; sugar substitutes, sweeteners, and all syrups (excluding 100% maple syrup); distilled alcoholic drinks

*Significant linear trend across all quintiles (*p* < 0.001), both in unadjusted and models adjusted for sex, age group (1-5, 6–11, 12–19, 20–39, 40–59, 60+ years), race/ethnicity (Mexican-American, Other Hispanic, Non-Hispanic White, Non-Hispanic Black and Other Race – Including Multi-Racial), ratio of family income to poverty (SNAP 0.00–1.30, >1.30–3.50, and >3.50 and over), and educational attainment (<12, 12 years, and >12 years)

We then compared the average dietary content of macronutrients (expressed as percent of total energy) and of micronutrients and fiber (both expressed as g/1,000 kcal) across quintiles of dietary share of ultra-processed foods.

Principal Component Analysis (PCA) is one of the methods that can be used to empirically derive dietary patterns. This is a mathematical technique that allows reducing the complexity of interrelationships among observed variables into a smaller number of uncorrelated linear combinations of them referred to as “components” and which maximize the explained variance [19, 42].

Using PCA, through the correlation matrix applied to the dietary content of macronutrients, micronutrients, and fiber, we identified four nutrient dietary patterns in the sample (Vitamin E was excluded because it loaded on all main extracted components). The four patterns were selected based on the Kaiser criterion (eigenvalue > 1.0), scree plot, and PCA components interpretability. The components were rotated using the varimax procedure and a factor score was calculated for each of the four patterns.

PCA was conducted in the whole sample and stratifying by age (1–5, 6–11, 12–19, 20–39, 40–59, 60+ years), sex, race/ethnicity (Mexican-American, Other Hispanic, Non-Hispanic White, Non-Hispanic Black, Other Race), ratio of family income to poverty line (0.00–1.30, >1.30–3.50, and >3.50) [32] and educational attainment of respondents aged 20+ years or of household reference person otherwise (<12, 12 years, and >12 years). Final PCA results are presented for all strata combined because, despite some variations, comparable patterns were observed across sociodemographic strata.

We used Gaussian regression to estimate the association between the dietary share of ultra-processed foods and the four component factor scores. To relax the linearity assumption of the association, the dietary contribution of ultra-processed foods variable was transformed using restricted cubic splines with five knots. The model was also fit using z-standardized scores. The factor scores were then regressed on the quintiles of the dietary share of ultra-processed foods. Finally, factor scores were categorized into tertiles to express *low*, *middle,* and *high* adherence to the dietary pattern in order to examine the category distribution across quintiles of the dietary share of ultra-processed foods.

All regression models were adjusted for age, sex, race/ethnicity, family income [32], and educational attainment. As 908 participants had missing values on family income and/or educational attainment, adjusted analyses included 8,409 individuals.

NHANES survey sample weights were used in all analyses except the PCA correlation matrix, to account for differential probabilities of selection for the individual domains, nonresponse to survey instruments, and differences between the final sample and the total US population. The Taylor series linearization variance approximation procedure was used to account for complex sample design and sample weights [32]. Tests of linear trend were performed to evaluate the effect of quintiles as a single continuous variable.

To minimize chance findings from multiple comparisons, statistical hypotheses were tested using a two-tailed *p* ≤ 0.001 level of significance. Data were analyzed using Stata version 12.1.

## Results

### Distribution of total energy intake according to food groups and across quintiles of dietary share of ultra-processed foods

The average US daily energy intake in 2009–2010 was 2,069.9 kcal, 57.5% of calories coming from ultra-processed foods, 30.2% from unprocessed or minimally processed foods, 9.3% from processed foods and 2.9% from processed culinary ingredients (Table 1). The energy contribution of most subgroups belonging to ultra-processed foods increased monotonically from the first to the last quintile of the dietary share of ultra-processed foods, with a few exceptions that showed a slight decrease between the fourth and fifth quintiles. An opposite trend was observed among subgroups from all three remaining groups.

### Nutrient dietary contents according to dietary share of ultra-processed foods

The average dietary protein content decreased significantly and monotonically across quintiles of the dietary share of ultra-processed foods (from 17.9% of total energy intake in the lowest quintile to 13.1% in the highest). The content of alcohol evolved in a similar way (from 4.1% to 0.9% of total energy intake). In contrast, across the same quintiles, there were significant increases in the content of carbohydrates (from 46.5% to 53.4%), added sugars (7.7% to 19.2%), and saturated fats (10.1% to 10.9%) (Table 2).

**Table 2:** Indicators of the dietary content of macronutrients and micronutrients according to the dietary share of ultra-processed foods, US population aged 1+ years (NHANES 2009–2010) (*N =* 9,317)

		Quintiles of dietary share of ultra-processed foods (% of total energy intake) [n]^a^				
		Q1 [*n =* 1,941]	Q2 [*n =* 1,903]	Q3 [*n =* 1,791]	Q4 [*n =* 1,785]	Q5 [*n =* 1,897]
Macronutrient Indicators (mean % of total energy intake)	Protein	17.9	16.7	15.8	14.7	13.1*
	Total carbohydrates	46.5	48.6	49.9	51.3	53.4*
	Added sugars	7.7	11	13.4	15.7	19.2*
	Total fats	31.4	32.2	32.5	32.6	32.5
	Saturated fats	10.1	10.7	10.9	10.9	10.9*
	Alcohol	4.1	2.4	1.8	1.4	0.9*
Micronutrient Indicators (mean density)	Fiber (g/1,000 kcal)	9.6	8.9	8.2	7.4	6.7*
	Sodium (g/1,000 kcal)	1.74	1.69	1.69	1.66	1.63
	Vitamin A (μg/1,000 kcal)	377.5	358.5	347.4	306.2	272.3*
	Vitamin C (mg/1,000 kcal)	58.2	51.4	42.9	40.3	32.4*
	Vitamin D (μg/1,000 kcal)	3.3	3.2	2.9	2.5	2.0*
	Vitamin E (mg/1,000 kcal)	4.1	3.8	3.6	3.5	3.3*
	Iron (mg/1,000 kcal)	7.4	7.7	7.8	7.5	7.4
	Zinc (mg/1,000 kcal)	6.3	6	5.8	5.4	4.9*
	Potassium (g/1,000 kcal)	1.6	1.4	1.3	1.2	1.0*
	Phosphorus (mg/1,000 kcal)	728.9	715.9	691.7	653.9	605.9*
	Magnesium (mg/1,000 kcal)	173.3	156.6	144.3	130.6	117.3*
	Calcium (mg/1,000 kcal)	531.1	539.6	532.2	507	464.7*

^a^Mean (range) dietary share of ultra-processed foods per quintile: 1st = 32.6 (0 to 42.6); 2nd = 48.6 (42.6 to 54.0); 3rd = 58.4 (54.0 to 62.8); 4th = 67.3 (62.8 to 72.3); 5th = 80.7 (72.3 to 100)

*Significant linear trend across all quintiles (*p* ≤ 0.001), both in unadjusted and models adjusted for sex, age group (1-5, 6–11, 12–19, 20–39, 40–59, 60+ years), race/ethnicity (Mexican-American, Other Hispanic, Non-Hispanic White, Non-Hispanic Black and Other Race – Including Multi-Racial), ratio of family income to poverty (SNAP 0.00–1.30, >1.30–3.50, and >3.50 and over), and educational attainment (<12, 12 years, and >12 years).

The average dietary content of fiber and of all micronutrients except iron and sodium decreased significantly and monotonically across quintiles of the dietary share of ultra-processed foods: fiber (from 9.6 in the lowest quintile to 6.7 g/1,000 kcal in the highest), vitamin A (377.5 to 272.3 μg/1,000 kcal), vitamin C (58.2 to 32.4 mg/1,000 kcal), vitamin D (3.3 to 2.0 μg/1,000 kcal) and vitamin E (4.1 to 3.3 mg/1,000 kcal), zinc (6.3 to 4.9 mg/1,000 kcal), potassium (1.6 to 1.0 g/1,000 kcal), phosphorus (728.9 to 605.9 mg/1,000 kcal), magnesium (173.3 to 117.3 mg/1,000 kcal), and calcium (531.1 to 464.7 mg/1,000 kcal). The sodium dietary content decreased non-significantly across quintiles of the dietary share of ultra-processed foods (from 1.74 to 1.63 g/1,000 kcal), while the iron content increased between the first and third quintiles and decreased thereafter.

### Nutrient dietary patterns obtained through PCA

Through PCA, four of 15 components had an eigenvalue >1.0 and explained 67% of the variance, and all four were retained. The rotated factor loadings of these four components are displayed in Table 3 (factor loadings above 0.20 and below -0.20 have been highlighted).

**Table 3:** Rotated factor loadings for the first four components from principal component analysis using nutrients, US population aged 1+ years (NHANES 2009–2010) (*N =* 9,317)

	PC1	PC2	PC3	PC4
Indicator^a^	(% expl.^b^ = 20.4)	(% expl. = 18.0)	(% expl. = 17.7)	(% expl. = 10.9)
Fiber density (g/1,000 kcal)	**0.47** ^**c**^	-0.12	0.00	0.09
Sodium density (g/1,000 kcal)	0.04	**-0.22**	**0.39**	**0.20**
Potassium density (mg/1,000 kcal)	**0.44**	0.15	0.10	-0.08
Iron density (mg/1,000 kcal)	0.02	0.00	-0.09	**0.68**
Zinc density (mg/1,000 kcal)	-0.08	0.06	0.16	**0.53**
Phosphorus density (mg/1,000 kcal)	0.09	**0.38**	**0.21**	0.08
Magnesium density (mg/1,000 kcal)	**0.44**	0.05	0.11	0.05
Calcium density (mg/1,000 kcal)	0.02	**0.55**	-0.07	0.02
Vitamin A density (μg/1,000 kcal)	0.06	**0.24**	-0.09	**0.24**
Vitamin C density (mg/1,000 kcal)	**0.40**	0.07	-0.15	**-0.21**
Vitamin D density (μg/1,000 kcal)	-0.02	**0.55**	-0.08	0.00
Protein (% of total energy)	0.05	0.03	**0.45**	0.14
Carbohydrate (% of total energy)	0.17	0.00	**-0.54**	0.17
Added sugars (% of total energy)	**-0.24**	-0.03	**-0.41**	0.14
Saturated fat (% of total energy)	**-0.34**	**0.30**	**0.22**	-0.16

^a^For details on indicators, see Methods section

^b^Proportion of the variance explained by each factor after orthogonal varimax rotation (Kaiser on)

^c^Items with a factor loading above 0.20 or below -0.20 have been highlighted using boldface

The first component was characterized by being richer in fiber, potassium, magnesium, and vitamin C, and having less saturated fat and added sugars (variables with factor loadings above 0.20 or below -0.20). The factor loading for sodium was close to zero in this first component (0.04). This component, called *nutrient balanced pattern*, was selected as an instrument to measure the quality of the diet overall.

Each of the three remaining components mixed healthy and unhealthy features regarding dietary nutrient contents. The second component indicated higher content in both saturated fat and micronutrients such as calcium, vitamin D, phosphorus, and vitamin A and lower content in sodium. The third showed higher content in protein, saturated fat, and sodium and phosphorus, and lower content in carbohydrates and added sugars. The fourth presented higher content in iron, zinc, vitamin A and sodium, and lower in vitamin C.

Comparable PCA patterns were observed across sociodemographic strata. This was especially true for the *Nutrient balanced pattern* as illustrated for race/ethnicity strata in Additional file 1: Table S2.

### Association between the dietary share of ultra-processed foods and the nutrient balanced pattern

**Fig. 1:**
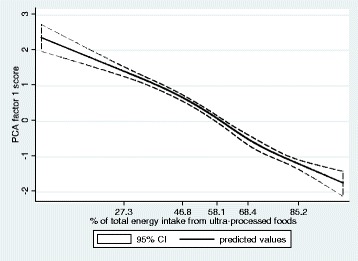
“Nutrient balanced pattern” factor score regressed on the dietary share of ultra-processed foods evaluated by restricted cubic splines, US population aged 1+ years (NHANES 2009–2010) (*N =* 9,317). Legend: The values shown on the x-axis correspond to the 5th, 27.5th, 50th, 72.5th, and 95th percentiles for percentage of total energy from ultra-processed foods (knots). Coefficient for linear term = -0.03, 95% CI: -0.04 to -0.02 (beta = -0.35). There was little evidence of nonlinearity in the restricted cubic spline model (Wald test for linear term *p* < 0.001; Wald test for all non-linear terms *p* = 0.16)

In unadjusted restricted cubic splines Gaussian regression analysis, a strong linear association was identified between the dietary share of ultra-processed foods and the *nutrient balanced pattern* factor score (coefficient for linear term = -0.03, 95% CI: -0.04 to -0.02) (Fig. 1). There was little evidence of nonlinearity in the restricted cubic spline model (Wald test for linear term *p* < 0.001; Wald test for all non-linear terms *p* = 0.16). The strength of the association remained nearly the same after adjusting for sex, age, race/ethnicity, family income, and educational attainment (coefficient for linear term = -0.04, 95% CI: -0.05 to -0.03). According to the adjusted model, one standard deviation increase in the dietary share of ultra-processed foods leads to a 0.38 standard deviation decrease in the *nutrient balanced pattern* factor score.

Across quintiles of the dietary share of ultra-processed foods, the adjusted mean *nutrient balanced pattern* factor score decreased monotonically, from 1.1 in the lowest quintile to -0.9 in the highest **(**Table 4 **)**. Across the same quintiles, the proportion of individuals with *high* adherence to the *nutrient balanced pattern* decreased monotonically from 58.4% in the lowest quintile of the dietary share of ultra-processed foods to 11.0% in the highest. Inversely, the proportion of individuals with *low* adherence increased from 13.3% in the lowest quintile to 61.7% in the highest (overall Chi square test *p* < 0.001).

**Table 4:** “Nutrient balanced pattern” factor score means and adherence according to the dietary share of ultra-processed foods, US population aged 1+ years (NHANES 2009–2010)

Dietary share of ultra-processed foods (% of total energy intake)		“Nutrient balanced pattern” factor score		Adherence to “Nutrient balanced pattern”^b^		
Quintiles	Mean (range)	Mean		Low (%)	Middle (%)	High (%)
		unadj. (R2 = 0.18)	adj.^a^ (R2 = 0.24)			
Q1 (*n =* 1,941)	32.6 (0 to 42.6)	1.2*	1.1*	13.3	28.3	58.4
Q2 (*n =* 1,903)	48.6 (42.6 to 54.0)	0.6*	0.5*	19.6	35.0	45.5
Q3 (*n =* 1,791)	58.4 (54.0 to 62.8)	0.04	0.002	30.0	37.3	32.7
Q4 (*n =* 1,785)	67.3 (62.8 to 72.3)	-0.5*	-0.4*	42.2	38.8	19.0
Q5 (*n =* 1,897)	80.7 (72.3 to 100)	-1.0*^¥^	-0.9*^¥^	61.7	27.4	11.0

^a^Adjusted for sex, age group (1-5, 6–11, 12–19, 20–39, 40–59, 60+ years), race/ethnicity (Mexican-American, Other Hispanic, Non-Hispanic White, Non-Hispanic Black and Other Race – Including Multi-Racial), ratio of family income to poverty (SNAP 0.00–1.30, >1.30–3.50, and >3.50 and over), and educational attainment (<12, 12 years, and >12 years)

^b^“Nutrient balanced pattern” (PC1) factor score tertiles: T1 (-4.7 to -0.9 points); T2 (-0.9 to 0.6 points); T3 (0.6 to 9.9 points)

*Statistically significant *p* ≤ 0.001

^¥^Significant linear trend across all quintiles (*p* ≤ 0.001), both in unadjusted and models adjusted for sex, age group, race/ethnicity, ratio of family income to poverty, and educational attainment

The dietary share of ultra-processed foods also presented an inverse association with the remaining three components (Additional file 1: Figure S1). The mean factor scores of these three remaining components also decreased across the dietary share of ultra-processed foods (Additional file 1: Table S3).

## Discussion

In this analysis of US nationally representative data, we show that a significant linear inverse relationship exists between the dietary contribution of ultra-processed foods and the dietary content of protein, fiber, vitamins A, C, D, and E, zinc, potassium, phosphorus, magnesium, and calcium. On the other hand, carbohydrate, saturated fat, and added sugar contents increased significantly with the dietary contribution of ultra-processed foods. Only diets in the lowest quintile of ultra-processed consumption had the average added sugar content below the upper limit recommended by the 2015–2020 Dietary Guidelines for Americans [40], while the average saturated fat content exceeded the same limit in all quintiles, with the lowest quintile moving closest to the recommendation.

We also found an inverse dose–response association between ultra-processed food dietary contribution and the overall dietary quality measured through a *nutrient balanced pattern* PCA-derived factor score characterized by being richer in fiber, potassium, magnesium, and vitamin C, and having less saturated fat and added sugars. Furthermore, we found substantially higher adherence to the *nutrient balanced pattern* in lower quintiles of ultra-processed food dietary contribution than in higher ones. These results are relevant because both individual education interventions and food environment regulatory policies have the potential to modify the dietary content of ultra-processed foods. To our knowledge, this is the first study to evaluate the association between the dietary contribution of ultra-processed foods and the overall nutritional quality of diets in the US.

The non-significant but somewhat unexpected sodium content decrease across quintiles of the dietary share of ultra-processed foods may be partly explained by the fact that in the US processed foods include basically “salty products” – such as cheese, ham, or vegetables in brine – while most ultra-processed foods are either “sweet products” (soft, fruit, and milk drinks, cakes, cookies, breakfast cereals, ice cream, sweet snacks, industrialized desserts) or products containing both salt and sugar (breads, sauces, canned soups, dressings, gravies, dips, spreads, mustard, catsup). Still, the sodium dietary content was above the Tolerable Upper Intake Level for any sex-age group [40] regardless of the share of ultra-processed foods.

The not uncommon iron fortification of ultra-processed foods or their ingredients may explain why the iron content does not show the reverse gradient across quintiles of ultra-processed food consumption seen among other micronutrients.

Few studies have assessed the impact of levels of food processing on the nutrient contents of the US diet. One study [43] that applied a food-industry-supported classification system [44] to NHANES 2003–2008 food intake data found that, together, “mixtures of combined ingredients” and “ready-to-eat,” which are mostly ultra-processed foods, contributed to 51% of total energy intake in the US diet but to only 37% of the protein intake and to 73% of the added sugar intake. These two food groups also contributed to 37% of the fiber intake and to between 30% and 60% of the intake of micronutrients [43]. Analyses of the same data restricted to children and adolescents [45] and to adults [46] showed similar results. Unfortunately, these studies on data from NHANES 2003–2008 failed to explore whether the dietary content of critical nutrients actually differed between high and low consumers of “mixtures of combined ingredients” plus “ready-to-eat.”

Another study evaluated US household barcoded purchasing data from 2000 to 2012 using a classification system guided by the one used in our study [12]. In 2012, the mean per capita purchase of “highly processed foods,” a category similar to ultra-processed foods, had higher adjusted median saturated fat, total sugar, and sodium content than “less processed foods.” This report did not capture non-barcoded items such as unpackaged fresh fruit, vegetables, and meat, or highly processed foods such as ready-to-eat store-prepared items, and did not explore whether the dietary content of critical nutrients actually differed between high and low consumers of “highly processed foods".

Consistent with our results, an investigation in Canada using 2001 household purchasing data found a decrease in protein content and fiber density across quintiles of the energy share of ultra-processed foods, together with an increase in the content of free sugars and total fats [10].

A study carried out in Brazil using 2008–2009 national food intake data found that protein, fiber, sodium, and potassium decreased significantly across quintiles of the dietary contribution of ultra-processed foods, while free sugars, total fats, and saturated fats increased [8]. After adjusting for family income, there was a significant drop in the dietary content of vitamin D, vitamin E, phosphorus, magnesium, and zinc, and an increase in calcium [9].

Our study has several strengths. We studied a large, nationally representative sample of the US population, increasing generalizability. Our investigation was based on total effective individual consumption data, rather than on household purchasing data [7, 10, 47], which do not evaluate the fraction of wasted food or purchases at restaurants.

Potential limitations should be considered. As with most population measures, dietary data obtained by 24-h recalls are imperfect. However, 24-h recalls are the least-biased self-report instrument available. Also, standardized methods and approach of NHANES have been shown to produce accurate intake estimates [33–35], and will therefore be suitable for assessing food group contributions and nutrient densities in the overall diet. Although NHANES collects limited information indicative of food processing (i.e., place of meals, product brands), these data are not consistently determined for all food items and this may lead to groups classification errors. Also, as some authors have highlighted, the number of food items reported in NHANES is smaller than the number available in the marketplace, and national food composition data are not updated as required to include all brand-specific products and to examine dietary profiles sensitive to brand preferences [48]. The PCA method also has limitations such as subjective decisions regarding the number of extracted components, method of rotation, naming of components, and cutoffs for factor loadings [23, 31, 49].

## Conclusions

This study suggests that decreasing the dietary share of ultra-processed foods is a rational and effective way to substantially improve dietary quality in the US.

## Additional file

Additional file 1: Table S1. Characteristics of study participants and of the full sample of interviewed participants aged 1 year and above, US population aged 1+ years (NHANES 2009–2010). **Table S2.** Rotated factor loadings for the first four components from principal component analysis using nutrients, across race/ethnicity strata, US population aged 1+ years (NHANES 2009–2010) (*N*=9,317). **Table S3.** PC2-PC4 score means and adherence according to the dietary share of ultra-processed foods, US population aged 1+ years (NHANES 2009–2010). **Figure S1.** PC2-PC4 factor scores regressed on the dietary share of ultra-processed foods evaluated by restricted cubic splines, US population aged 1+ years (NHANES 2009–2010) (*N*=9,317). (DOCX 1047 kb)

## Abbreviations

FNDDS: USDA food and nutrient database for dietary studies
FPED: Food patterns equivalents database
FPID: Food patterns equivalents ingredients database
INFORMAS: International network for food and obesity/non-communicable diseases research, monitoring and action support
NHANES: National health and nutrition examination survey
PCA: Principal component analysis
SR Codes: Standard reference codes
USDA: National nutrient database for standard reference, release 24 (SR24)
