# Supplementary material for: The share of ultra-processed foods and the overall nutritional quality of diets in the US: evidence from a nationally representative cross-sectional study
Source: Popul Health Metr. 2017 Feb 14;15:6. doi: 10.1186/s12963-017-0119-3 (PMC5307821; doi:10.1186/s12963-017-0119-3)
Supplement: Additional file 1: Table S1. — Characteristics of study participants and of the full sample of interviewed participants aged 1 year and above, US population aged 1+ years (NHANES 2009–2010). Table S2. Rotated factor loadings for the first four components from principal component analysis using nutrients, across race/ethnicity strata, US population aged 1+ years (NHANES 2009–2010) (N=9,317). Table S3. PC2-PC4 score means and adherence according to the dietary share of ultra-processed foods, US population aged 1+ years (NHANES 2009–2010). Figure S1. PC2-PC4 factor scores regressed on the dietary share of ultra-processed foods evaluated by restricted cubic splines, US population aged 1+ years (NHANES 2009–2010) (N=9,317). (DOCX 1047 kb) [file 12963_2017_119_MOESM1_ESM.docx]

Supplemental Table 1: Characteristics of study participants and of the full sample of interviewed participants aged 1 year and above, US population aged 1+ years (NHANES 2009–2010)

|  |  | Study (N=9,317) | Full sample (N=10,109) |
| --- | --- | --- | --- |
| Gender | Men | 49.0 | 48.9 |
|  | Women | 50.9 | 51.0 |
|  |  |  |  |
| Age (years) | 1 to 5 | 6.7 | 7.1 |
|  | 6 to 11 | 8.1 | 8.2 |
|  | 12 to 19 | 11.3 | 11.1 |
|  | 20 to 39 | 27.2 | 27.3 |
|  | 40 to 59 | 28.4 | 28.2 |
|  | 60 and over | 18.3 | 18.2 |
|  |  |  |  |
| Race/ethnicity | Mexican American | 10.4 | 10.4 |
|  | Other Hispanic | 5.4 | 5.4 |
|  | Non-Hispanic White | 65.2 | 64.8 |
|  | Non-Hispanic Black | 11.9 | 12.0 |
|  | Other Race (including Multi-Racial) | 7.2 | 7.4 |
|  |  |  |  |
| Income to poverty | 0.00–1.30 | 24.7 | 24.8 |
|  | >1.30–3.50 | 36.8 | 36.7 |
|  | >3.50 and above | 38.5 | 38.5 |
|  |  |  |  |
| Educational attainment | <12 years | 19.2 | 19.4 |
|  | 12 years | 22.6 | 22.7 |
|  | >12 years | 58.2 | 58.0 |

Supplemental Table 2: Rotated factor loadings for the first four components from principal component analysis using nutrients, across race/ethnicity strata, US population aged 1+ years (NHANES 2009–2010) (N=9,317)

|  | **Mexican-American** (n=2,064) | | | |  | **Other Hispanic** (n=988) | | | |
| --- | --- | --- | --- | --- | --- | --- | --- | --- | --- |
|  | PC1 | PC2 | PC3 | PC4 |  | PC1 | PC2 | PC3 | PC4 |
| Indicator^1^ | (% expl.^2^=21.7) | (% expl.=18.2) | (% expl.=17.6) | (% expl.=10.2) |  | (% expl.=21.2) | (% expl.=19.4) | (% expl.=16.6) | (% expl.=11.0) |
| Fiber density (g/1,000 kcal) | **0.49^3^** | -0.18 | 0.00 | 0.04 |  | **0.45** | -0.12 | -0.01 | 0.11 |
| Sodium density (g/1,000 kcal) | 0.00 | **-0.21** | **0.41** | 0.09 |  | 0.05 | **-0.24** | **0.31** | **0.26** |
| Potassium density (mg/1,000 kcal) | **0.43** | 0.18 | 0.05 | -0.05 |  | **0.46** | 0.13 | 0.11 | -0.14 |
| Iron density (mg/1,000 kcal) | -0.01 | -0.08 | -0.05 | **0.70** |  | 0.05 | 0.05 | -0.18 | **0.67** |
| Zinc density (mg/1,000 kcal) | -0.03 | 0.03 | **0.24** | **0.43** |  | -0.03 | 0.11 | 0.13 | **0.49** |
| Phosphorus density (mg/1,000 kcal) | 0.17 | **0.32** | **0.21** | 0.09 |  | 0.07 | **0.36** | **0.22** | 0.12 |
| Magnesium density (mg/1,000 kcal) | **0.48** | 0.00 | 0.10 | 0.01 |  | **0.42** | 0.09 | 0.09 | 0.05 |
| Calcium density (mg/1,000 kcal) | 0.05 | **0.54** | -0.09 | 0.03 |  | 0.00 | **0.53** | -0.04 | 0.02 |
| Vitamin A density (μg/1,000 kcal) | -0.03 | 0.16 | -0.07 | **0.45** |  | 0.12 | **0.31** | -0.12 | 0.13 |
| Vitamin C density (mg/1,000 kcal) | **0.34** | 0.13 | **-0.23** | -0.12 |  | **0.39** | 0.06 | -0.10 | **-0.31** |
| Vitamin D density (μg/1,000 kcal) | -0.02 | **0.54** | -0.08 | 0.08 |  | -0.03 | **0.53** | -0.05 | -0.05 |
| Protein (% of total energy) | 0.10 | 0.00 | **0.47** | 0.11 |  | 0.08 | -0.02 | **0.46** | **0.20** |
| Carbohydrate (% of total energy) | 0.18 | -0.06 | **-0.52** | 0.19 |  | **0.20** | 0.02 | **-0.55** | 0.11 |
| Added sugars (% of total energy) | **-0.25** | -0.10 | **-0.33** | 0.13 |  | **-0.24** | -0.01 | **-0.44** | 0.12 |
| Saturated fat (% of total energy) | **-0.32** | **0.37** | 0.19 | -0.12 |  | **-0.36** | **0.30** | **0.22** | -0.14 |

|  | **Non-Hispanic White** (n=3,984) | | | |  | **Non-Hispanic Black** (n=1,726) | | | |
| --- | --- | --- | --- | --- | --- | --- | --- | --- | --- |
|  | PC1 | PC2 | PC3 | PC4 |  | PC1 | PC2 | PC3 | PC4 |
| Indicator^1^ | (% expl.=20.1) | (% expl.=19.0) | (% expl.=18.0) | (% expl.=11.2) |  | (% expl.^2^=20.9) | (% expl.=18.5) | (% expl.=15.4) | (% expl.=11.4) |
| Fiber density (g/1,000 kcal) | **0.49** | -0.13 | -0.01 | 0.08 |  | **0.44** | 0.00 | -0.11 | 0.15 |
| Sodium density (g/1,000 kcal) | 0.04 | **-0.17** | **0.37** | **0.23** |  | 0.04 | **0.39** | **-0.23** | **0.22** |
| Potassium density (mg/1,000 kcal) | **0.43** | 0.14 | 0.12 | -0.07 |  | **0.46** | 0.10 | 0.13 | -0.08 |
| Iron density (mg/1,000 kcal) | 0.05 | 0.02 | -0.11 | **0.65** |  | 0.02 | -0.10 | -0.01 | **0.68** |
| Zinc density (mg/1,000 kcal) | -0.08 | 0.08 | 0.11 | **0.59** |  | -0.05 | 0.14 | 0.03 | **0.47** |
| Phosphorus density (mg/1,000 kcal) | 0.04 | **0.40** | **0.20** | 0.07 |  | 0.08 | **0.26** | **0.36** | 0.10 |
| Magnesium density (mg/1,000 kcal) | **0.43** | 0.05 | 0.09 | 0.06 |  | **0.44** | 0.13 | 0.04 | 0.03 |
| Calcium density (mg/1,000 kcal) | -0.02 | **0.55** | -0.04 | 0.01 |  | 0.03 | -0.07 | **0.57** | 0.04 |
| Vitamin A density (μg/1,000 kcal) | 0.16 | **0.31** | -0.09 | 0.07 |  | 0.04 | -0.07 | 0.12 | **0.34** |
| Vitamin C density (mg/1,000 kcal) | **0.42** | 0.06 | -0.12 | **-0.20** |  | **0.38** | -0.14 | 0.12 | **-0.26** |
| Vitamin D density (μg/1,000 kcal) | -0.05 | **0.55** | -0.07 | -0.04 |  | 0.01 | -0.08 | **0.57** | 0.05 |
| Protein (% of total energy) | 0.02 | 0.08 | **0.41** | 0.19 |  | 0.06 | **0.49** | -0.02 | 0.07 |
| Carbohydrate (% of total energy) | 0.10 | 0.07 | **-0.57** | 0.14 |  | **0.23** | **-0.51** | -0.03 | 0.16 |
| Added sugars (% of total energy) | **-0.24** | 0.01 | **-0.43** | 0.12 |  | **-0.20** | **-0.40** | -0.06 | 0.12 |
| Saturated fat (% of total energy) | **-0.31** | **0.23** | **0.28** | **-0.20** |  | **-0.38** | 0.18 | **0.33** | -0.05 |

|  | **Other Race - Including Multi-Racial** (n=555) | | | |
| --- | --- | --- | --- | --- |
|  | PC1 | PC2 | PC3 | PC4 |
| Indicator^1^ | (% expl.=20.5) | (% expl.=18.9) | (% expl.=17.9) | (% expl.=11.6) |
| Fiber density (g/1,000 kcal) | **0.41** | -0.08 | -0.07 | 0.12 |
| Sodium density (g/1,000 kcal) | 0.08 | **-0.24** | **0.40** | 0.13 |
| Potassium density (mg/1,000 kcal) | **0.47** | 0.11 | 0.14 | -0.08 |
| Iron density (mg/1,000 kcal) | -0.02 | 0.00 | -0.05 | **0.70** |
| Zinc density (mg/1,000 kcal) | **-0.23** | -0.01 | **0.30** | **0.46** |
| Phosphorus density (mg/1,000 kcal) | 0.08 | **0.39** | **0.25** | 0.03 |
| Magnesium density (mg/1,000 kcal) | **0.43** | 0.02 | 0.14 | 0.04 |
| Calcium density (mg/1,000 kcal) | 0.03 | **0.53** | -0.11 | 0.14 |
| Vitamin A density (μg/1,000 kcal) | 0.16 | **0.26** | -0.10 | **0.33** |
| Vitamin C density (mg/1,000 kcal) | **0.40** | 0.01 | -0.18 | -0.12 |
| Vitamin D density (μg/1,000 kcal) | 0.02 | **0.51** | -0.02 | -0.02 |
| Protein (% of total energy) | 0.02 | -0.01 | **0.54** | 0.04 |
| Carbohydrate (% of total energy) | 0.18 | -0.13 | **-0.43** | **0.24** |
| Added sugars (% of total energy) | **-0.27** | -0.03 | **-0.33** | 0.11 |
| Saturated fat (% of total energy) | **-0.26** | **0.39** | 0.03 | **-0.20** |

Supplemental Figure 1: PC2-PC4 factor scores regressed on the dietary share of ultra-processed foods evaluated by restricted cubic splines, US population aged 1+ years (NHANES 2009–2010) (N=9,317)

| **A.** Unadjusted model: There was little evidence of linearity in the restricted cubic spline model (Wald test for linear term p=0.8; Wald test for all non-linear terms p<0.001). Adjusted model: Wald test for linear term p=0.3; Wald test for all non-linear terms p=0.003.  **B.** Coefficient for linear term = -0.03, 95% CI: -0.04 to -0.01. There was little evidence of nonlinearity in the restricted cubic spline model (Wald test for linear term p=0.002; Wald test for all non-linear terms p=0.03). Adjusted model: Coefficient for linear term = -0.03, 95% CI: -0.04 to -0.01; Wald test for linear term p=0.001; Wald test for all non-linear terms p=0.02.  **C.** Unadjusted model: There was little evidence of linearity in the restricted cubic spline model (Wald test for linear term p=0.3; Wald test for all non-linear terms p=0.03). Adjusted model: Wald test for linear term p=0.1; Wald test for all non-linear terms p=0.02. |
| --- |

Supplemental Table 3: PC2-PC4 score means and adherence according to the dietary share of ultra-processed foods, US population aged 1+ years (NHANES 2009–2010)

| Dietary share of ultra-processed foods (% of total energy intake) | |  | “Calcium and Vitamin D driven pattern” factor score | |  | Adherence to “calcium and Vitamin D driven pattern”^2^ | | |  | “Protein driven pattern” factor score | |  | Adherence to “protein driven pattern”^3^ | | |  | “Iron and zinc driven pattern” factor score | |  | Adherence to “iron and zinc driven pattern”^4^ | | |
| --- | --- | --- | --- | --- | --- | --- | --- | --- | --- | --- | --- | --- | --- | --- | --- | --- | --- | --- | --- | --- | --- | --- |
|  |  |  | Mean | |  |  |  |  |  | Mean | |  |  |  |  |  | Mean | |  |  |  |  |
| Quintiles | Mean (range) |  | unadj. (R2=0.06) | adj.^1^ (R2=0.18) |  | Low (%) | Middle (%) | High (%) |  | unadj. (R2=0.16) | adj.^1^ (R2=0.20) |  | Low (%) | Middle (%) | High (%) |  | unadj. (R2=0.01) | adj.^1^ (R2=0.02) |  | Low (%) | Middle (%) | High (%) |
| Q1 (n=1941) | 32.6 (0 to 42.6) |  | 0.32* | 0.41* |  | 26.4 | 32.0 | 41.6 |  | 1.08* | 1.08* |  | 15.2 | 29.1 | 55.8 |  | 0.09 | 0.06 |  | 30.5 | 32.5 | 37.0 |
| Q2 (n=1903) | 48.6 (42.6 to 54.0) |  | 0.27* | 0.26* |  | 24.7 | 33.1 | 42.1 |  | 0.57* | 0.57* |  | 22.5 | 33.7 | 43.8 |  | 0.14 | 0.13 |  | 29.5 | 31.9 | 38.7 |
| Q3 (n=1791) | 58.4 (54.0 to 62.8) |  | 0.06 | 0.03 |  | 29.9 | 32.3 | 37.8 |  | 0.22 | 0.23 |  | 31.1 | 37.3 | 31.6 |  | 0.14 | 0.13 |  | 29.8 | 34.0 | 36.2 |
| Q4 (n=1785) | 67.3 (62.8 to 72.3) |  | -0.24* | -0.26* |  | 35.2 | 36.3 | 28.6 |  | -0.23* | -0.17 |  | 39.6 | 39.2 | 21.2 |  | -0.06 | -0.04 |  | 33.8 | 36.7 | 29.5 |
| Q5 (n=1897) | 80.7 (72.3 to 100) |  | -0.69*¥ | -0.71*¥ |  | 50.5 | 32.9 | 16.6 |  | -0.83*¥ | -0.76*¥ |  | 58.3 | 27.5 | 14.2 |  | -0.16¥ | -0.12 |  | 43.2 | 31.6 | 25.2 |

^1^Adjusted for sex, age group (1-5, 6–11, 12–19, 20–39, 40–59, 60+ years), race/ethnicity (Mexican-American, Other Hispanic, Non-Hispanic White, Non-Hispanic Black, and Other Race – Including Multi-Racial), ratio of family income to poverty (SNAP 0.00–1.30, >1.30–3.50, and >3.50 and over), and educational attainment (<12, 12 years, and >12 years).

^2^“Calcium and Vitamin D driven pattern” (PC1) factor score tertiles: T1 (-4.6 to -0.8 points); T2 (-0.8 to 0.3 points); T3 (0.3 to 9.8 points).

^3^“Protein driven pattern” (PC1) factor score tertiles: T1 (-8.9 to -0.5 points); T2 (-0.5 to 0.8 points); T3 (0.8 to 8.4 points).

^4^“Iron and zinc driven pattern” (PC1) factor score tertiles: T1 (-4.5 to -0.6 points); T2 (-0.6 to 0.3 points); T3 (0.3 to 12.9 points).

*Statistically significant p<=0.001

^¥^Significant linear trend across all quintiles (p<=0.001), both in unadjusted and models adjusted for sex, age group, race/ethnicity, ratio of family income to poverty, and educational attainment.
